# Regulation of Extracellular Matrix Production in Activated Fibroblasts: Roles of Amino Acid Metabolism in Collagen Synthesis

**DOI:** 10.3389/fonc.2021.719922

**Published:** 2021-08-27

**Authors:** Emily J. Kay, Grigorios Koulouras, Sara Zanivan

**Affiliations:** ^1^Cancer Research UK Beatson Institute, Glasgow, United Kingdom; ^2^Institute of Cancer Sciences, University of Glasgow, Glasgow, United Kingdom

**Keywords:** extracellular matrix, fibroblasts, CAF, tumour microenvironment, metabolism, amino acids

## Abstract

Cancer associated fibroblasts (CAFs) are a major component of the tumour microenvironment in most tumours, and are key mediators of the response to tissue damage caused by tumour growth and invasion, contributing to the observation that tumours behave as ‘wounds that do not heal’. CAFs have been shown to play a supporting role in all stages of tumour progression, and this is dependent on the highly secretory phenotype CAFs develop upon activation, of which extracellular matrix (ECM) production is a key element. A collagen rich, stromal ECM has been shown to influence tumour growth and metastasis, exclude immune cells and impede drug delivery, and is associated with poor prognosis in many cancers. CAFs also extensively remodel their metabolism to support cancer cells, however, it is becoming clear that metabolic rewiring also supports intrinsic functions of activated fibroblasts, such as increased ECM production. In this review, we summarise how fibroblasts metabolically regulate ECM production, focussing on collagen production, at the transcriptional, translational and post-translational level, and discuss how this can provide possible strategies for effectively targeting CAF activation and formation of a tumour-promoting stroma.

## Introduction

Fibroblasts are one of the most abundant cell types in the microenvironment of solid tumours, and have long been known to play multiple and varied roles in promoting tumour progression and metastasis. Fibroblasts are influenced by tumour cells to become ‘activated’, a process during which they develop a highly secretory phenotype involving production of growth factors, pro-angiogenic factors, immunomodulatory factors, metabolites, extracellular vesicles, and, crucially, ECM components and remodelling factors (1–5). Activated fibroblasts in the tumour microenvironment are known as cancer associated fibroblasts, or CAFs, however, fibroblasts undergo an extremely similar activation process during wound healing, or other fibrotic diseases (6, 7). Indeed, the role of activated fibroblasts is vital in the process of wound healing to stimulate cell proliferation, blood vessel repair and formation, immune cell recruitment to prevent infection and ECM production to provide structural support for wound closure. However, following wound healing, fibroblasts revert to their quiescent state whereas in cancer or fibrotic disease, fibroblasts are aberrantly and continuously activated, leading to the description of tumours as ‘wounds that do not heal’ (8).

**Figure 1:**
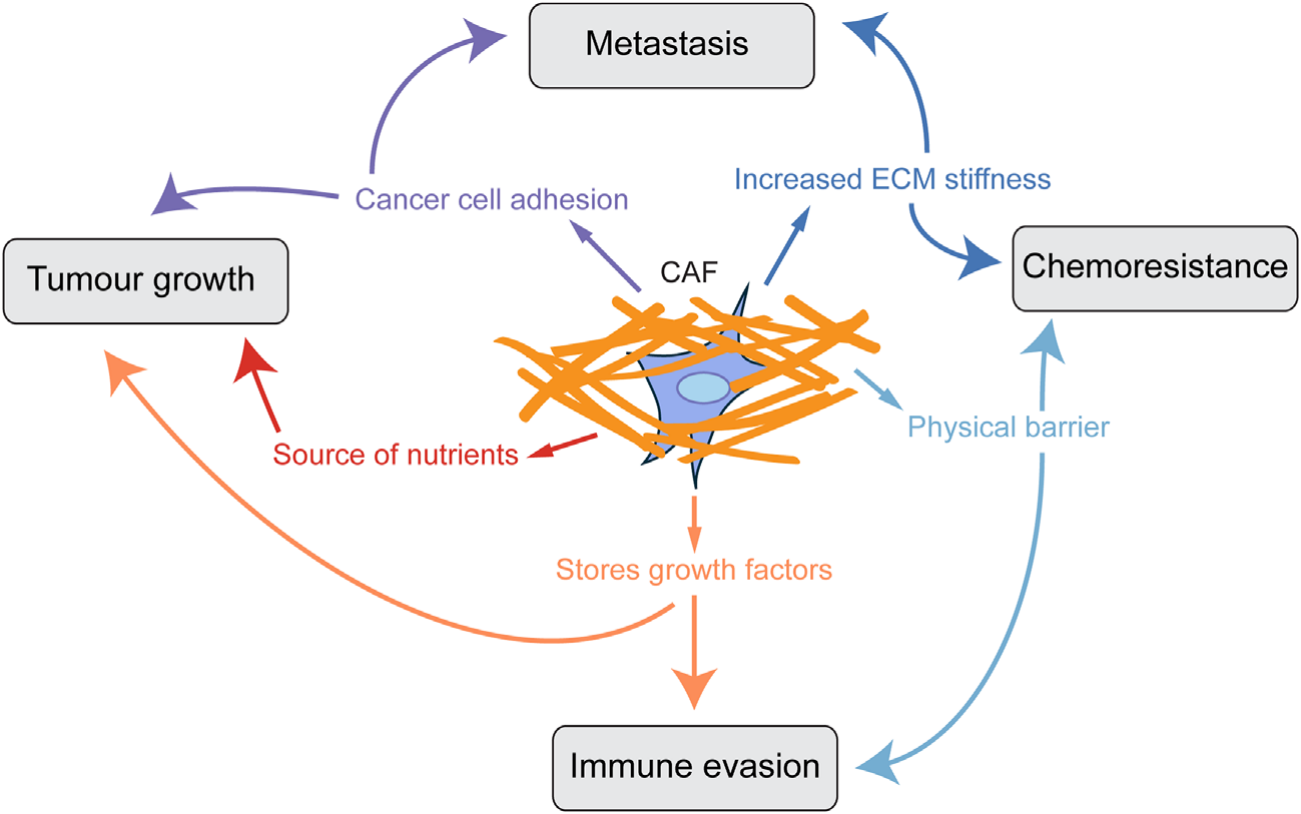
CAF-derived ECM promotes tumour progression. Scheme showing how a collagen-rich ECM produced by CAFs influences key aspects of tumour progression.

One of the main roles of fibroblasts in the healthy body is to produce and maintain turnover of the extracellular matrix (ECM), of which collagen proteins are one of the most highly abundant components, and indeed comprise approximately 30% of the total protein content of mammals (9). Upon fibroblast activation, however, production of ECM and collagen is vastly upregulated. In cancer, the production of excessive collagen-rich ECM by CAFs is a crucial step in tumour progression, and CAFs are the main source of structural ECM in tumours (10, 11). Studies have shown that a collagen-dense stromal compartment is a predictor of poor prognosis in many cancer types (12–14). ECM provides a substrate for integrin-mediated signalling supporting cancer cell adhesion and proliferation (15–18), acts as a reservoir of pro-angiogenic and growth factors, can be degraded to provide amino acids for tumour cells (19, 20) and also acts as a physical barrier to decrease tumour perfusion, drug delivery and infiltration of tumour suppressing immune cells (21, 22). Furthermore, collagen in the tumour microenvironment is more heavily cross-linked and linearised, leading to a stiffer ECM which is also known to increase tumour aggression (23, 24). The remodelling of the ECM and linearization of collagen fibres is an important step in the deposition of pro-tumorigenic ECM, since non-linearised collagen I can be anti-tumourigenic (25). *In vivo*, the effects of the ECM on tumour growth have been assessed in several studies. Ablation of Col6a1 or Col5a3 in the MMTV-PyMT mammary tumour model resulted in reduced hyperplasia and primary tumour growth (26, 27). Conversely, mice which have been engineered to produce more collagen (Col1a1^tm1jae^) showed increased tumour growth in the MMTV-PyMT model (12, 28). Inhibition of production of other ECM components such as hyaluronan, fibronectin and tenascin-C also suppresses tumour initiation and growth (29, 30). Therefore finding ways to target ECM production by CAFs could both reduce tumour growth and metastasis and improve tumour perfusion and drug delivery (**Figure 1**) (29–33).

## Energetic Cost of ECM Production

**Figure 2:**
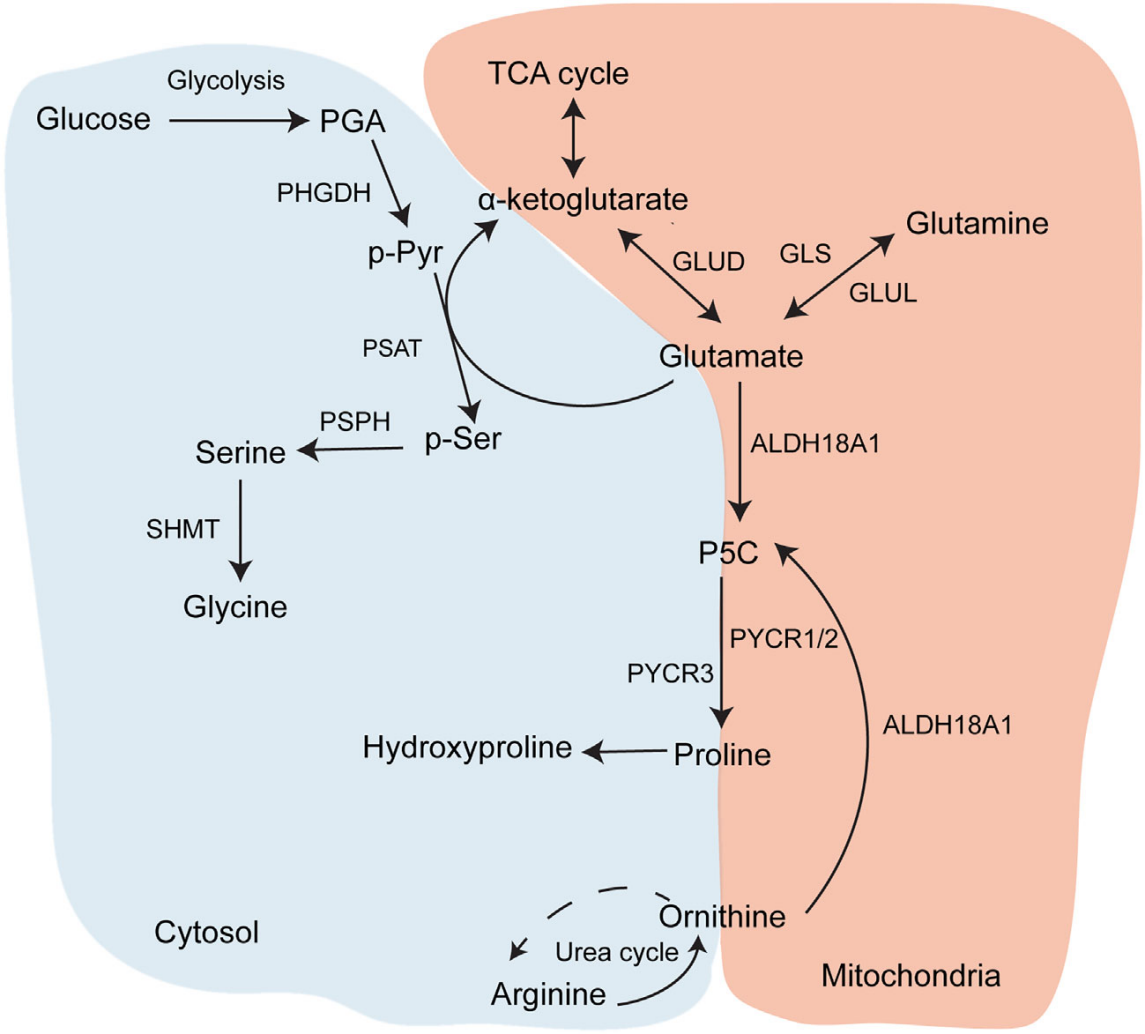
Glycine and proline biosynthesis. Metabolic pathways contributing to synthesis of proline and glycine, which are the two most abundant amino acids in collagen.

Of all the proteins that make up the ECM, collagen has a particularly unusual amino acid composition. The collagen protein is composed primarily of the Gly-X-Y motif, in which X and Y are most commonly proline and its modified form hydroxyproline (34). This is because small, flexible amino acids are required to fit into the helix conformation of collagen chains, and in particular glycine is the only amino acid small enough to fit into the centre of the triple helix. Furthermore, the hydroxyproline residues can form hydrogen bonds along the helix to stabilise it. As a result, collagens contain approximately 30% glycine and 15-20% proline or hydroxyproline residues, although this varies between different collagens (**Table 1**). Therefore, collagen synthesis has unique biosynthetic requirements and, given that it is a major output of CAFs, it is expected that they might remodel their metabolism accordingly to sustain it. Both proline and glycine are non-essential amino acids, and can therefore be obtained exogenously from blood or made endogenously. Glycine is the smallest amino acid and its production from larger molecules is actually an exogenic process. Glycine is produced from serine and its synthesis is therefore connected to the tetrahydrofolate cycle and to glycolysis, both of which produce ATP (**Figure 2**). Proline synthesis, on the other hand, is an ATP-consuming process. Proline can be synthesised either from glutamine *via* conversion to glutamate, or from arginine *via* conversion to ornithine. Synthesis of 1 mole proline from 1 mole glutamine or arginine requires 8 or 2.5 moles ATP, respectively (35). The arginine pathway for proline synthesis therefore has the least energetic cost.

**Table 1:** A list of collagens with the percentage of glycine and proline residues in each.

Collagen Type	Proline content (%)	Glycine content (%)
Collagen I	17.5	27.3
Collagen II	18.1	27.3
Collagen III	18.7	28.1
Collagen IV	21.7	27.7
Collagen V	17.6	24.8
Collagen VI	8.2	11.6
Collagen VII	14.4	21.2
Collagen VIII	22.4	26.5
Collagen IX	16.6	27.9
Collagen X	21.3	25.7
Collagen XI	16.7	24.0
Collagen XII	9.2	9.2
Collagen XIII	17.4	25.6
Collagen XIV	8.7	10.8
Collagen XV	13.8	15.9
Collagen XVI	17.5	24.3
Collagen XVII	13.6	18.8
Collagen XVIII	17.3	17.2

Specific amino acid requirements aside, the increased production of ECM proteins has a more general energetic cost. Structural proteins such as collagens and fibronectin have a high number of amino acid residues, meaning that their translation is costly in terms of ATP and GTP. Post-translational modification of proline to hydroxyproline also has a high energetic cost, with 4 mole of ATP required to produce 1 mole of hydroxyproline for collagen synthesis. Once translated and modified, ECM proteins are then secreted by exocytosis, itself an ATP-consuming process (36).

Although it has long been known that tumour cells undergo metabolic alterations, only in the last decade has the remodelling of CAF metabolism been studied in detail. Most studies focus on the role of CAF metabolism in supporting tumour cell proliferation through secretion of metabolites such as lactate, pyruvate, and amino acids. Increased glycolysis and autophagy are the two mechanisms most commonly observed in CAF metabolic rewiring in the context of CAFs providing metabolites to fuel tumour cells (37–43). However, until recently there has been little research into how CAFs rewire their metabolism to support their own needs upon activation, and in particular to support ECM production. In addition to data available on how activated fibroblasts metabolically support ECM production in cancer, we can look to research on ECM production by fibroblasts in wound healing and fibrosis for further insights, since these behave similarly to CAFs (44). Indeed, studies show that there are metabolic similarities between CAFs and other types of activated fibroblasts, such as increased glycolysis (45–47).

## Glycolysis Stimulates ECM Production in Fibroblasts

Increased TGFβ signalling is the most well-known and studied hallmark and master regulator of fibroblast activation, both in cancer and in other fibroblast activating conditions. Among its many roles, TGFβ signalling upregulates production of ECM, including collagens, in activated fibroblasts (48, 49). TGFβ has also been linked to metabolic reprogramming in CAFs; in particular, it has been shown to upregulate glycolysis in many studies. Although previous work has focussed on the role of TGFβ-induced glycolysis in CAFs in producing lactate as a metabolic fuel for tumour cells, termed the ‘Reverse Warburg effect’ (38, 50), it is important to note that glycolysis is also the major source of ATP production in cells. As discussed earlier, the process of ECM production is ATP consuming, through requirements for specific amino acids, protein translation and post-translational modification. Since ECM is such a significant output of CAFs, it is reasonable to predict that an increase in glycolysis may also support ECM synthesis *via* increased ATP production. In support of this, it has been shown that fibroblasts require an increase in glucose uptake and glycolysis to support TGFβ-induced collagen production in fibrosis (51, 52).

Glucose metabolism is also required for the synthesis of glycine, the most abundant amino acid in collagen, and therefore increased TGFβ-induced glycolysis in CAFs could also support collagen production through providing precursors for glycine synthesis. Although glycine is available exogenously (~400 µM in plasma) (53), two studies have demonstrated that TGFβ signalling also increases serine and glycine synthesis in activated fibroblasts. Nigdegliogou and co-workers (51) demonstrated that the enzymes for serine and glycine synthesis from glucose, PDGDH and SHMT2 (**Figure 2**), were upregulated in TGFβ-treated human lung fibroblasts, in addition to glycolytic enzymes. Pharmacological inhibition or genetic deletion of PDGDH and SHMT2 both attenuated TGFβ-induced collagen I production. Since glycolysis provides precursors for both glycine and serine synthesis (**Figure 2**), this implies that upregulated glycolysis in activated fibroblasts can also be used to fuel glycine biosynthesis, which is a requirement for collagen production. The mechanism for TGFβ-stimulated glycine production was further elucidated by Selvarajah and co-workers (54), who recently demonstrated that, in human lung fibroblasts, canonical TGFβ signalling through SMAD3 activated mTORC1 enhanced expression of glycine synthesis enzymes and the glucose transporter GLUT1 *via* upregulation of the transcription factor ATF4. Inhibition of this pathway reduced glycine incorporation into, and thereby production of, collagen I. A further study by Woodcock and co-workers (55) also found that pharmacological inhibition of the mTORC1/4EBP1 signalling pathway attenuated collagen I synthesis in TGFβ-treated human lung fibroblasts and in CAFs derived from lung adenocarcinoma patients. mTOR signalling was also found to be upregulated in CAFs isolated from human PDAC tumours, although its effect on ECM production was not investigated (56). These studies show firstly that there is a requirement for increased amino acid production to support collagen synthesis, and also suggest a further role for TGFβ-induced glycolysis and mTOR signalling in activated fibroblasts to support glycine synthesis for collagen production. The role of mTOR signalling in this pathway is also of interest, as mTORC1 has long been known to be regulated by availability of amino acids (57), including glutamine which is involved in collagen production. Therefore it is also possible that when amino acids are available in activated fibroblasts, activated mTOR signalling regulates transcription of genes involved in metabolic pathways that promote collagen synthesis, both through increasing ATP production *via* glycolysis and further synthesis of specific amino acids required for translation of collagen mRNA.

## Proline Synthesis is Required for Collagen Production in Activated Fibroblasts

Collagen synthesis has often been hypothesised to be a metabolic ‘dump’ for excess proline. Both glutamine and arginine can be converted into 1-pyrroline-5-carboxylic acid (P5C) (*via* ALDH18A1 or OAT), which is the final precursor for proline synthesis by PYCR1, PYCR2 or PYCR3. It seems clear that proline synthesis is upregulated in activated fibroblasts and a limiting factor in collagen production. Hepatic stellate cells increase proline production from glutamine upon activation during liver fibrosis, and PYCR1 is upregulated and proline oxidase (PRODH), which recycles proline back to P5C, is downregulated, showing that fibroblast activation pushes cells towards proline synthesis, although whether this affected collagen production was not investigated (58). A recent study showed that TGFβ-activated fibroblasts increased expression of genes in the proline synthesis pathway as well as increasing proline labelling from ^13^C-glutamine. ALDH18A1 deletion decreased collagen production, which could be rescued with proline supplementation (52). PYCR1 deletion did not however affect collagen synthesis. Conversely, PYCR1 knockdown or inhibition reduced collagen production, and particularly collagen VI production, in patient derived mammary CAFs, and could be rescued with proline supplementation (59). P5C supplementation has also been shown to increase collagen synthesis by human fibroblasts (60). Furthermore, *Pycr1* KO zebrafish have reduced ECM content and proline and hydroxyproline levels in their tissues, demonstrating a link between proline availability and ECM production (61). Interestingly, mutations in *PYCR1* or *ALDH18A1* in patients give rise to a condition called cutis laxa, one of the symptoms of which is wrinkled skin. This could be due to a loss of ECM production by fibroblasts, and indeed abnormal collagen fibres and decreased collagen compactness, in addition to reduced elastin content, has been observed in some patients with *PYCR1* mutations (62). Reduced levels of collagens I and III has also been observed in patients with *ALDH18A1* mutations (63).

In addition to proline biosynthesis, extracellular proline is a potential source of proline for collagen synthesis. Several studies have investigated the effects of extracellular proline on collagen production in fibroblasts. Proline concentration is upregulated at wound sites, suggesting it is either actively imported to or synthesised at the wound and therefore there may be a requirement for extracellular proline (64). However, an early study found that proline supplementation does not increase collagen production in a range of cell lines in culture, although fibroblasts were not investigated (65). Although cirrhotic rat liver contains high levels of proline and collagen, a proline rich diet did not stimulate collagen production in the liver, suggesting the high proline concentration comes from proline synthesis (66). More recent research, including from our lab, has confirmed this observation (59). Proline supplementation did not increase collagen production in human mammary CAFs and human skin fibroblasts unless glutamine availability or proline synthesis was limited (59, 67). Furthermore, although exogenous proline increased Col1a1 expression and radiolabelled proline was incorporated into collagen in human dermal fibroblasts, this effect was more pronounced when the fibroblasts were cultured in the absence of glutamine (67). Therefore, it seems that fibroblasts preferentially synthesise their own proline. The study on dermal fibroblasts proposed that proline availability also regulates expression of collagen genes as well as being a substrate for collagen translation, which suggests there could be a feedback loop whereby intracellular proline concentration regulates collagen expression. However, this was not the case in the mammary CAFs, so this may not be a universal mechanism for activated fibroblasts.

The question of why fibroblasts prefer to synthesise their own proline for collagen production, even when free proline is available, has not yet been answered. One possibility is that proline synthesis plays an important role in producing reducing potential. The production of proline by PYCR1 oxidises NAPDH or NADH to NADP+/NAD+, which can support glycolysis and the pentose phosphate pathway (68), which could help to maintain the increase in glycolysis in activated fibroblasts. Equally, the interconversion of P5C and proline creates a shuttle of the redox equivalents NADPH/NADP+ between the mitochondria and cytosol, meaning that proline production can play a role in maintaining redox homeostasis (69). Proline itself is also an antioxidant through the secondary amine of the pyrrolidine ring (70). In support of this, upregulated proline synthesis protects cells from the reducing potential and ROS caused by increased TCA cycle activity in TGF-β stimulated fibroblasts (52), and mitochondrial NADPH was required for proline biosynthesis and collagen production in MEFs (71). PYCR1 loss in fibroblasts has been shown to increase their susceptibility to ROS-mediated apoptosis (72). Interestingly, both PYCR1 and PYCR2 have been found to interact with and promote the activity of RRM2B, a protein that supports DNA damage repair in response to oxidative stress, in fibroblasts, showing that the anti-oxidant properties of PYCR1 are not solely due to its role in proline production but that it also plays a role in the wider cellular response to oxidative stress (73). Therefore fibroblasts may also maintain proline synthesis to counteract redox stress. Thus, in addition to reducing ECM production, targeting collagen production in CAFs through proline synthesis could also reduce their ability to cope with the increased levels of oxidative stress in the tumour microenvironment, and further research into the effects of proline synthesis inhibition on CAFs would be needed to verify this.

## Precursors for Proline Synthesis

### Glutamine Metabolism

Another major metabolic pathway, which has been found to regulate ECM production in fibroblasts, and in particular collagen production, is that of glutamine metabolism. Glutamine is converted to glutamate, and from there can enter the TCA cycle *via* α-ketoglutarate to fuel oxidative phosphorylation. Glutamate is also a precursor for proline. Intraperitoneal administration of glutamine improved wound healing and increased the presence of immature collagen in parenchymal lung lesions in rats (74). Interestingly, dietary glutamine supplementation improved collagen density in colonic anastomoses in rats more than glycine supplementation (75), suggesting that fibroblasts are able to synthesise sufficient glycine for collagen production, whereas they require a source of extracellular glutamine. This is also reflected in a study showing that a much higher concentration of extracellular glycine was needed to increase collagen production in chondrocytes than that of glutamine or, indeed, proline (76). Conversely, inhibition of glutamine metabolism with the glutamine agonist 6-diazo-5-oxo-L-norleucine (DON) prevented aspects of fibrosis including collagen production in fibroblasts derived from patients with iatrogenic laryngotracheal stenosis (iLTS) (77). Conversion of glutamine into glutamate seems to be crucial for its collagen-promoting properties, as inhibition of glutamate synthase (GLS) with the inhibitor BPTES also decreased collagen production in iLTS derived fibroblasts (78). Furthermore, both glutamine and glutamate stimulated collagen biosynthesis in human skin fibroblasts (60), and glutamine synthesis has been shown to be upregulated in ovarian CAFs (79). However, although these studies show that glutamine metabolism is important for collagen production both *in vivo* and in activated fibroblasts *in vitro*, the question remains as to whether glutamine enhances collagen production through incorporation into proline to sustain collagen translation, or through other metabolic pathways leading indirectly to increased collagen expression, or both.

A few studies have demonstrated that glutamine is required for proline production to sustain collagen synthesis in fibroblasts. Bellon and co-workers (80) first demonstrated that glutamine supplementation stimulates procollagen synthesis in human foreskin fibroblasts, and that glutamine-derived proline competed with extracellular ^14^C-labelled proline for incorporation into prolyl-tRNA and procollagen, showing that glutamine is an important intracellular source of proline for collagen production. Furthermore, procollagen synthesis was independent of the concentration of free proline in the media when glutamine was provided, suggesting that fibroblasts may prefer to synthesise their own proline from glutamine rather than use extracellular proline. A more recent study showed that conversion of glutamine into glutamate and thence to proline and glycine (**Figure 2**) is required for collagen production in human lung fibroblasts activated with TGFβ (81). TGFβ increased the expression of GLS, PSAT1 and enzymes in the proline synthesis pathway, and the intracellular concentrations of both proline and glycine, in addition to increasing collagen production. In the absence of glutamine, collagen production, but not *COL1A1* mRNA expression, was reduced, implying again that glutamine is required for collagen translation. siRNA mediated silencing of GLS, PSAT1 or ALDH18A1 attenuated TGFβ-induced collagen production, and interestingly ALDH18A1 knockdown could not be rescued by proline supplementation at physiological levels, suggesting again that proline synthesis from glutamine, rather than extracellular proline, is required for collagen synthesis in activated fibroblasts. This is concurrent with data showing only supraphysiological levels of proline could rescue PYCR1 knockdown (59), however another study was able to rescue ALDH18A1 depletion with sub physiological proline levels (52). This could be because the CRISPR mediated ALDH18A1 knockout in Schworer et al. has a more drastic effect on proline synthesis compared to the siRNA and shRNA knockdown in the other two studies, and can therefore a lower dose of proline will provide some rescue. Conversely, inhibition of glutamate metabolism by oxoglutarate dehydrogenase knockdown to decrease oxidative metabolism did not affect collagen production.

### Arginine and Ornithine

Aside from glutamine, cells can also make proline for collagen production from arginine *via* ornithine, a pathway which branches from the urea cycle (**Figure 2**). Much of the evidence that arginine metabolism supports collagen production comes from studies on wound healing and fibrosis, however there is some evidence that this pathway may be similarly regulated in CAFs. Glutamate, arginine and ornithine are all drained at burn sites (82, 83), suggesting a requirement for these specific amino acids during the wound healing process. Furthermore, arginase expression is upregulated in wound derived fibroblasts at all stages of the wound healing process in rats (84) and local inhibition of arginase delayed healing of incisional wounds in C57Bl/6 mice (85). Arginine is also among the metabolites upregulated in the lungs of patients with idiopathic lung fibrosis (86), although the study did not investigate whether the increase in arginine levels was specifically in fibroblasts or in other cells in the lung. In an immunohistochemical analysis of PDAC patients, arginase has been found to be upregulated in CAFs, and is a predictor of poor outcome. Furthermore, arginase expression could be stimulated in cultured fibroblasts by exposure to hypoxia, which is a common feature of the tumour microenvironment (87). Therefore arginine and ornithine metabolism seems to be upregulated in conditions in which fibroblasts increase collagen production. But does it actually contribute to collagen synthesis? Dietary supplementation of both arginine and ornithine, but not citrulline, has been shown to improve collagen production in wounds in mice or rats (88–90). Furthermore, arginase is upregulated in fibroblasts in mice treated with bleomycin to stimulate fibrosis, and pharmacological inhibition of arginase with NG-hydroxy-l-arginine attenuated TGFβ-stimulated collagen deposition, without affecting collagen mRNA expression or SMAD signalling, suggesting that arginine is required for collagen translation, possibly through conversion to proline (91). Also linking TGFβ-induced collagen deposition by fibroblasts to arginase activity is a study showing that treating rats given lung orthotopic transplants with pirfenidone reduced collagen content and fibro-collagenous injury in the transplants, and that this was associated with both decreased endogenous TGFβ and arginase expression (92). TGFβ was also shown to stimulate arginine uptake and ornithine aminotransferase (OAT) expression in smooth muscle cells (93). Arginine-induced collagen production by smooth muscle cells was found to be dependent on conversion of arginine to proline (94), suggesting arginine’s role in collagen synthesis is as a proline precursor. However, much of the research into arginine metabolism and collagen production has been done in the context of wound healing and fibrosis, and while activated fibroblasts in wounds and the TME share similarities, further research is required to verify whether targeting arginase also reduces collagen production in tumours. Arginase has already been proposed as a therapeutic target against tumour promoting immune cells, so if it also stimulates collagen production in CAFs it could be a useful means of targeting two aspects of the tumour microenvironment.

## Alternative Roles for Amino Acid Metabolism in Collagen Production

**Figure 3:**
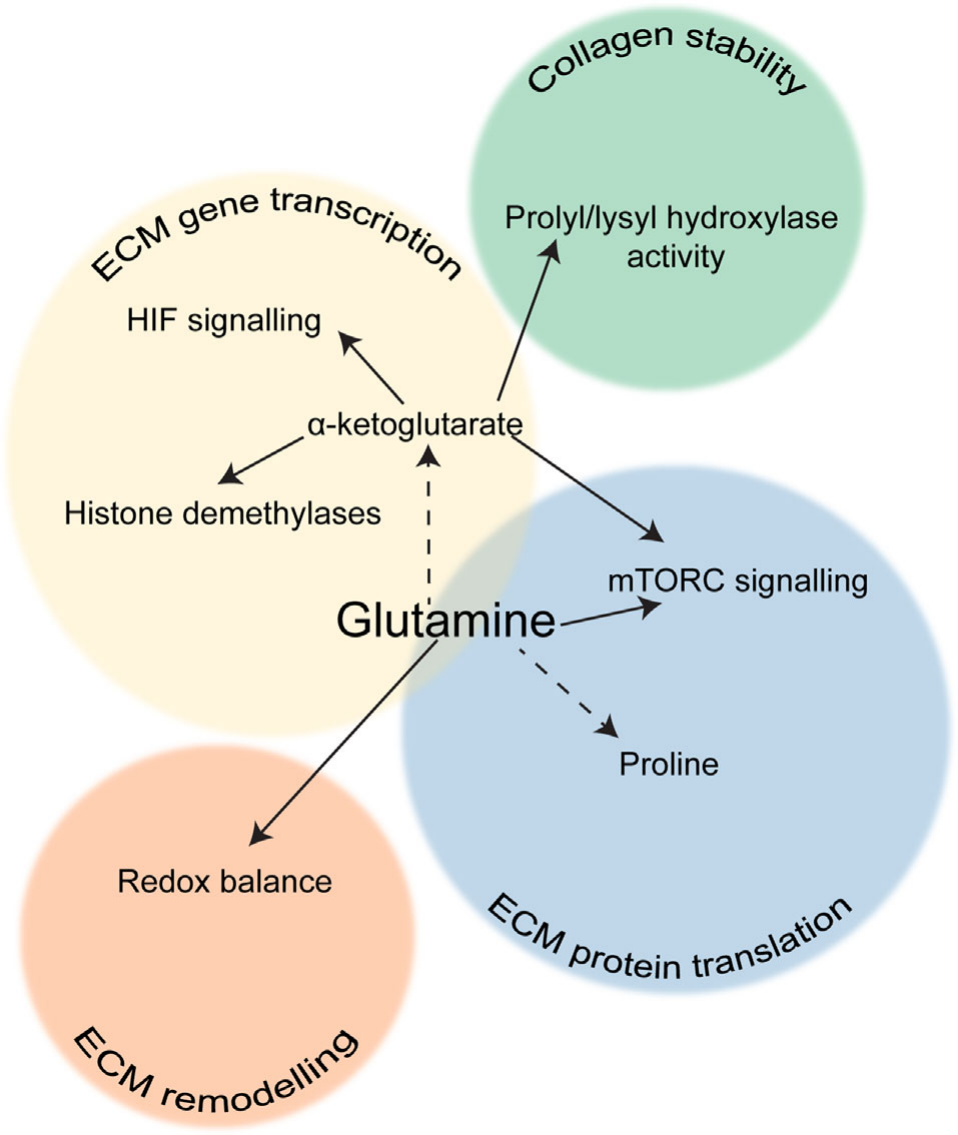
The role of glutamine in ECM production. Scheme showing the different aspects of ECM production that are influenced by glutamine metabolism in fibroblasts.

Many studies have shown that the role of glutamine metabolism in collagen synthesis is not limited to the translational level (**Figure 3**). Interestingly, glutamine availability can also regulate collagen mRNA expression in fibroblasts. Treatment of cultured fibroblasts with glutamine increased collagen mRNA levels (95), conversely, removal of glutamine from cell culture media or pharmacological inhibition of GLS reduced expression of collagen I in hepatic stellate cells (58). It has also been shown that glutamine metabolism may regulate fibroblast activation at a more general level. Bernard and co-workers found that when murine lung fibroblasts are deprived of glutamine or GLS is inhibited, TGFβ treatment fails to increase not only the expression of *Col1a1*, but also other markers of fibroblast activation including fibronectin, *Acta2* (which encodes for αSMA) and *Hif1a*. Interestingly, the authors also found that glutamine depletion post-TGFβ treatment did not affect αSMA protein levels but did affect the other markers, and that α-ketoglutarate only restored *Acta2* and *Hif1a* expression under glutamine deprivation (96). On the other hand, another study showed that in human lung fibroblasts GLS inhibition did not affect collagen gene expression, but reduced collagen translation *via* loss of mTORC1 activation, which was regulated by α-ketoglutarate production from glutamate (97). This therefore suggests that glutamine metabolism may support fibroblast activation through several different pathways. It is worth noting that α-ketoglutarate is a cofactor for many enzymes, including histone demethylases, so it is possible that glutamine metabolism may promote expression of *Acta2* and *Hif1a* through an epigenetic switch, whereas perhaps expression of ECM proteins is part of a feedback loop regulated by amino acid availability. Conversion of α-ketoglutarate to succinate could also inhibit prolyl hydroxylases that destabilise HIF1α.

An alternative way that glutamine metabolism can affect collagen production is through α-ketoglutarate-mediated activation of prolyl hydroxylases, which use it as a cofactor. Conversely, accumulation of succinate decreases prolyl hydroxylase activity. Proline hydroxylation is vital to maintain collagen stability, and GLS inhibition markedly increased degradation of collagens I and III in human lung fibroblasts (97). The requirement for α-ketoglutarate by prolyl hydroxylases has also been linked to amino acid sensing by mTOR, since α-ketoglutarate is a degradation product of several amino acids and the product of glutamine deamination (98). As previously discussed, mTOR activation has also been linked to collagen production through activation of glycine synthesis and glycolysis. A more recent study demonstrated that HIF1α activation in chondrocytes led to increased glutaminolysis and thereby accumulation of α-ketoglutarate. This enhanced proline and lysine hydroxylation on collagen, making the matrix more resistant to degradation by MMPs and ultimately resulting in skeletal dysplasia (99). Since HIF1α signalling is also often activated in CAFs (100, 101), this mechanism could also be relevant for increased collagen modification in the tumour microenvironment. The α-ketoglutarate: succinate ratio has also been shown to affect collagen stability in breast CAFs (102). Another aspect of glutamine metabolism is its effect on the redox balance of the cell, as glutamate is a precursor for GSH synthesis, and it has also been shown that glutamine or cancer cell-derived glutamate balances the redox state of fibroblasts, enabling ECM remodelling and increased ECM stiffness (103). Therefore glutamine metabolism clearly has a wider impact on CAF-derived ECM than solely the translation of ECM proteins. Although it is clear that glutamine metabolism has an important role to play in ECM production by CAFs, the mechanism(s) by which it promotes fibrosis are unclear and studies are conflicting as to whether glutamine affects both collagen transcription and translation. More research is needed to determine exactly how glutamine metabolism can regulate mRNA expression of collagen, fibronectin and other myofibroblast markers. Therefore the exact role of glutamine in fibroblast activation and ECM production has yet to be determined, and it seems likely that glutamine metabolism impacts upon many pathways that can affect ECM gene expression, synthesis and stability.

Arginine metabolism may also play a more complex role in collagen production besides that of a proline precursor. Arginine is involved in the production of nitric oxide (NO), which has been shown in several studies to inhibit fibrosis and collagen production by fibroblasts (104–106). Therefore arginase and NO synthase (NOS) may compete for arginine as a substrate, and metabolism of arginine by arginase may divert arginine away from NO production in addition to enabling proline synthesis to stimulate collagen synthesis. On the other hand, NO production has actually been found upregulated in breast CAFs due to downregulation of Caveolin 1, which binds and inhibits NOS. NO production led to increased glycolysis and ROS production, both features of CAF activation (107). Interestingly, arginine supplementation was unable to enhance wound healing and collagen production in inducible NOS knockout mice (108), whereas ornithine supplementation still stimulated collagen production even in the absence of NOS (90). Therefore, while proline production from arginine and ornithine can be upregulated in activated fibroblasts to stimulate collagen production, whether or not arginine uptake regulates collagen production by reducing NO synthesis is still unclear and further research is required to elucidate the role of NO in fibroblast activation.

## Conclusions

TGFβ is well-known as a master regulator of CAF activation, but it is becoming clear that it is also a major architect of metabolic rewiring in fibroblasts. TGFβ stimulates glycolysis, serine and glycine metabolism, glutamine metabolism, and increased proline synthesis from glutamine and arginine. Therefore, in addition to increasing ECM gene expression, TGFβ also activates metabolic pathways that support ECM production by activated fibroblasts: by increasing ATP generation to support synthesis of ECM protein, increasing production of amino acids required for collagen translation and by enhancing collagen stability and post-translational modification.

Glycine, proline, glutamine and arginine metabolism are all potential targets for normalising collagen production in the tumour stroma to reduce tumour growth and improve tumour perfusion and drug delivery. However, there is still much research to be done and many unanswered questions. Firstly, many of the studies showing these metabolites affect collagen production have been carried out in the context of activated fibroblasts in wound healing, fibrotic disease and acutely TGFβ-treated fibroblasts, rather than CAFs derived from cancer patients, although gene expression data suggests these pathways are also upregulated in the tumour stroma. Therefore, further research is needed to verify that metabolic changes in CAFs are relevant and targetable pathways to regulate ECM production. Furthermore, there is a lack of studies investigating whether these metabolic pathways could be a viable therapeutic target against the tumour stroma *in vivo*, since the majority of research to date has focussed on the role of these pathways in 2D cell culture of activated fibroblasts and the only *in vivo* models have been of wound healing or fibrotic disease.

Therapeutically, there are already several possibilities for targeting metabolic regulation of ECM production in CAFs. The GLS inhibitor CB-839 is currently undergoing clinical trials in cancer patients, so it would be useful to ascertain whether this drug affects the stroma as well as targeting cancer cells. Arginase inhibitors are also available and undergoing clinical trials in cancer patients as an immunotherapeutic, again, it will be interesting to discover if they also have an impact on CAFs. However, the development of drugs targeting the proline synthesis pathway is still at an early stage, and inhibitors against PYCR1 have only recently been developed (109, 110). Since however, PYCR1 has recently been found to be upregulated in many cancer cells and to have tumour promoting effects both in cancer cells and CAFs (59, 111, 112), the development of new inhibitors may prove useful in targeting both tumour and stroma, killing two birds with one stone. Targeting stromal collagen production may also increase the effectiveness of immunotherapies. Collagen was shown to impede immune cell filtration, increase T-cell exhaustion and decrease sensitivity to PD-L1 blockade in lung tumours (113), although this may be tumour context dependent since in a *KRas*-induced PDAC mouse model, ablation of stromal collagen I enabled recruitment of tumour suppressing myeloid cells and promoted tumour progression (114). Finally, since normalisation of the tumour stroma is known to increase drug delivery to the tumour, it is likely that targeting the metabolic pathways discussed in this review will be most efficacious in combination with other cancer cell targeting therapies, and this should be borne in mind when designing future therapeutic strategies to target cancer-associated stroma and ECM production.

## Author Contributions

Conceived the work: EJK and SZ. Writing the manuscript: EJK. Generated data for Figure: GK. All authors contributed to the article and approved the submitted version.

## Funding

This work was funded by Cancer Research UK (A29800 to SZ) and Breast Cancer Now (2019AugPR1307 to SZ).

## Conflict of Interest

The authors declare that the research was conducted in the absence of any commercial or financial relationships that could be construed as a potential conflict of interest.

## Publisher’s Note

All claims expressed in this article are solely those of the authors and do not necessarily represent those of their affiliated organizations, or those of the publisher, the editors and the reviewers. Any product that may be evaluated in this article, or claim that may be made by its manufacturer, is not guaranteed or endorsed by the publisher.
